# Narrowing the Gap: Preserving Repertoire Diversity Despite Clonal Selection during the CD4 T Cell Response

**DOI:** 10.3389/fimmu.2015.00413

**Published:** 2015-08-11

**Authors:** Julia Merkenschlager, George Kassiotis

**Affiliations:** ^1^Mill Hill Laboratory, The Francis Crick Institute, London, UK; ^2^Department of Medicine, Faculty of Medicine, Imperial College London, London, UK

**Keywords:** CD4 T cell, TCR repertoire, TCR affinity, T cell response, clonotypic diversity

## Abstract

T cell immunity relies on the generation and maintenance of a diverse repertoire of T cell antigen receptors (TCRs). The strength of signaling emanating from the TCR dictates the fate of T cells during development, as well as during the immune response. Whereas development of new T cells in the thymus increases the available TCR repertoire, clonal selection during the immune response narrows TCR diversity through the outgrowth of clonotypes with the fittest TCR. To ensure maintenance of TCR diversity in the antigen-selected repertoire, specific mechanisms can be envisaged that facilitate the participation of T cell clonotypes with less than best fit TCRs. Here, we summarize the evidence for the existence of such mechanisms that can prevent the loss of diversity. A number of T cell-autonomous or extrinsic factors can reverse clonotypic hierarchies set by TCR affinity for given antigen. Although not yet complete, understanding of these factors and their mechanism of action will be critical in interventional attempts to mold the antigen-selected TCR repertoire.

## Introduction

Adaptive immunity provides formidable defense against an antigenically unpredictable array of infectious microbes and transformed cells. Recognition of diverse antigens relies on the generation and maintenance of a correspondingly diverse repertoire of antigen receptors, generated somatically and distributed clonally on lymphocytes (1).

Both chains of the heterodimeric αβ T cell receptor (TCRαβ, referred to here as TCR) are generated by a recombinatorial process of variable (V) and junctional (J), and in the case of the TCR β chain, also diversity (D), gene segment rearrangement. This process can create a conservatively estimated diversity of 10^15^ different TCRs (1), which recent studies suggest may be even higher (2–5). However, owing to the finite size of the T cell compartment, far fewer TCRs will ever be made in the life-time of an individual and even fewer will be successfully selected in the thymus. For example, TCR diversity in the mouse has been estimated in the order of 2 × 10^6^, a number that may be considered relatively small (5, 6). Nevertheless, this “practical” or “realized” TCR diversity appears sufficient to ensure that most, if not all, individuals will respond to most, if not all, antigens, a response perhaps facilitated by the cross-reactive nature of the TCR. Indeed, the mouse TCR repertoire contains on average ~100 naïve CD4^+^ T cells that bind a given antigenic peptide-MHC II (pMHCII) tetramer, most of which represent distinct clonotypes, each with a unique TCR (7–9). These different TCRs that share antigen reactivity are likely to recognize antigenic pMHCII complexes in different ways, receiving varying degrees of TCR signal strength.

The intrinsic ability of each TCR to recognize antigenic pMHCII complexes is largely responsible for the disparate behavior of distinct CD4^+^ T cell clonotypes during the immune response, both in terms of expansion (10–12) and T helper subset differentiation (13). This disparity, in turn, underlies the enrichment of the TCR repertoire in clonotypes that display the optimum TCR signal strength under the specific conditions. Indeed, fast outgrowth of clonotypes receiving the strongest TCR signal has been observed during CD4^+^ T cell priming in various systems (9, 14–18).

In addition to its involvement during priming, the available evidence suggests that TCR signal strength contributes to clonotypic selection during memory formation and recall responses. Several studies have demonstrated selection for CD4^+^ T cell clonotypes with stronger pMHCII tetramer binding or functional avidity during memory formation in response to immunization or infection (14, 19–22). In contrast, increased amount or potency of a given immunizing antigen (23, 24) or different models of infection or immunization (25–27) have been shown to favor lower-affinity CD4^+^ T cell clonotypes for entry into the memory pool. Moreover, individual CD4^+^ T cells transferred in separate hosts exhibit substantial variance in clonal expansion, even if they express identical TCRs (9). Thus, although these studies collectively support a role for TCR signal strength in clonotypic selection during both priming and memory formation, they also highlight the potential influence of factors other than TCR affinity.

Which parameters of immunization or infection can affect TCR clonotypic composition independently of TCR affinity, and indeed to what degree they can overcome TCR affinity-based hierarchies, remains poorly understood. It is clear, however, that such parameters have the potential to reduce the gap in TCR signal strength between high- and low-affinity clonotypes. This property may be critical in ensuring the necessary clonotypic diversity in the CD4^+^ T cell response. Here, we focus on CD4^+^ T cells (as the significant amount of knowledge on CD8^+^ T cells is reviewed elsewhere) and review the current knowledge of the factors that can modify the response of distinct T cell clonotypes that would otherwise be set by TCR affinity.

## A T Cell’s Behavior Altered by Other T Cells

The cell-autonomous effect of intrinsic TCR affinity on CD4^+^ T cell clonal expansion and selection is often taken to imply that CD4^+^ T cells responding to antigen are oblivious to other CD4^+^ T cells, including those with shared reactivity. However, the behavior of a given CD4^+^ T cell may be strongly affected by other CD4^+^ T cells, either through competition or regulation.

### Competition between clonotypes with shared reactivity

The advent of TCR-transgenic T cells permitted the artificial increase of the precursor frequency of T cell clones reactive with a given antigen. At unphysiologically high precursor frequencies, memory development of antigen-specific monoclonal CD4^+^ T cells was found to be severely compromised due to intraclonal competition (28–31).

Starting at more physiological precursor frequencies, monoclonal CD4^+^ T cells responding with relatively low functional avidity to an H2-A^b^-restricted epitope from the lymphocytic choriomeningitis virus (LCMV) glycoprotein (GP), failed to enter the memory pool when the GP epitope was expressed recombinantly in *Listeria monocytogenes*, but successfully competed with the host response following LCMV infection (32). The results of this study indicate that the fate of low-avidity monoclonal LCMV-specific CD4^+^ T cells, and their ability to compete with other clonotypes, is not simply intrinsically determined, but context-dependent.

In another TCR-transgenic system, low-avidity CD4^+^ T cell clonotypes in a semipolyclonal TCRβ-transgenic population reactive with an epitope from the surface glycoprotein (SU), encoded by the envelope (*env*) gene of Friend murine leukemia virus (F-MLV) were outcompeted by high-avidity clonotypes if they all were present even at low frequencies (18). However, removal of the high-avidity competitors permitted the full expansion of the low-avidity F-MLV-reactive CD4^+^ T cell clonotypes (18). Thus, the behavior of low-avidity CD4^+^ T cell clonotypes is modified by the relative composition of the antigenic pMHCII-reactive pool, and a given low-avidity CD4^+^ T cell will either clonally expand or not, depending on the absence or presence, respectively, of higher-avidity competitors.

**Figure 1:**
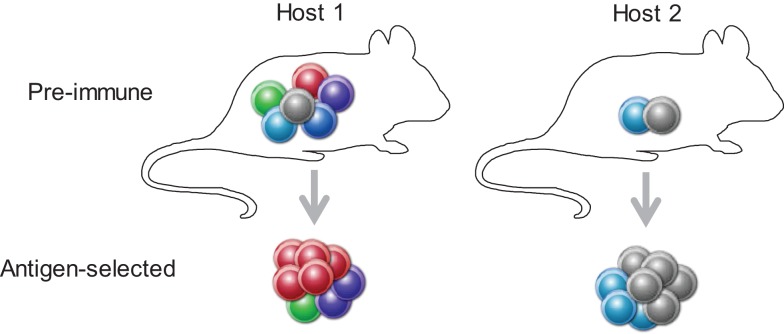
**A T cell’s behavior depends on the preimmune repertoire composition**. A given clonotype (depicted in gray) with relatively low TCR affinity for the immunizing antigen will either fail to expand (in the presence of higher affinity competitors, Host 1), or participate in the response (when competition is weaker, Host 2).

Competition between clonotypes with shared antigen reactivity may drive even more extreme differences in outcome if we consider the composition of the antigen-naïve TCR repertoire. Although current estimates suggest that reactivity for a given antigenic pMHCII complex is shared by, on average, 100 CD4^+^ T cells in the preimmune repertoire, this number varies substantially for different antigenic pMHCII complexes in the same individual from a theoretical one to experimentally observed several hundred (8). Moreover, owing to the random nature of repertoire formation, the preimmune pool of CD4^+^ T cells reactive with a given antigenic pMHCII complex will also vary between individuals, both in terms of numbers and clonotypic composition. For, example, the same low-avidity CD4^+^ T cell may have to compete with only one other similarly low-avidity CD4^+^ T cells in one individual, but with five other higher-avidity CD4^+^ T cells in another individual, and therefore, its behavior is expected to vary accordingly (Figure 1).

Although CD4^+^ T cell clonotypes with shared ability to recognize a particular antigenic pMHCII complex will compete for access to it, there are a number of additional factors that have been proposed to determine the outcome of clonotypic competition. These include multiple costimulatory factors, such as members of the B7 or TNF families of cytokines and costimulatory molecules and their receptors (33–35). In principle, limiting availability of costimulatory signals would render T cells more reliant on antigenic signals for their expansion, thus amplifying the advantage of those clonotypes that are more sensitive to antigen. Indeed, costimulation of virus-specific CD8^+^ T cells by CD27 was shown to permit the recruitment of lower-affinity clonotypes, promoting diversity in the response (36). A role for CD27 in maintaining clonotypic diversity in the CD4^+^ T cell response has not been directly demonstrated, but is supported by findings linking loss of CD27 expression in antigen-reactive effector CD4^+^ T cells with shortened half-life and inability to persist as memory (37). However, given the complex regulation of immune responses by both the B7 and TNF families, the overall effect of a particular costimulatory or inhibitory pathway on the clonotypic composition of a T cell response is often difficult to predict. For example, inclusion of multiple costimulatory pathways in the design of vaccine vectors was shown to favor higher affinity CD8^+^ T cells, rather than permitting recruitment of lower affinity CD8^+^ T cells (38). Moreover, engagement of the inhibitory molecule CTLA-4 was shown to increase the breadth of the CD4^+^ T cell response to immunization, likely by increasing, rather than decreasing clonotypic diversity (39). Nevertheless, these studies further support the notion that the particular constellation of costimulatory or inhibitory pathways seen in infection or immunization help shape the clonotypic diversity of antigen-reactive T cells.

Competition at the level of costimulation operates among T cell clonotypes with shared antigen reactivity, but may not be restricted to them. Expression of the receptors for many costimulatory or inhibitory molecules is typically induced on effector CD4^+^ T cells (33–35), and it is possible that through modulation of these pathways, T cells responding to a particular antigen affect other T cells concurrently responding to an unrelated antigen. Examples include depletion of costimulatory ligands on APCs by CTLA-4 on effector CD4^+^ T cells, thus depriving other effector CD4^+^ T cells of essential costimulation through CD28, the shared receptor for these ligands (40). Competition among CD4^+^ T cells irrespective of antigen specificity is akin to “quorum sensing,” a density-dependent mechanism that can adjust T cell numbers and maintain T cell homeostasis (41, 42). Although some evidence for “quorum sensing” operating at the T cell population level has been provided (41, 42), to what degree it determines the clonotypic hierarchy in the response to a particular antigen is not currently known.

### Clonotypic molding by regulatory T (Treg) cells

The preimmune CD4^+^ T cell pool also contains a significant proportion of regulatory T (Treg) cells, characterized by the expression of the transcription factor Foxp3, and naturally endowed with suppressive activity (43). Although originally thought to suppress autoimmune T cells, their suppressive activity against T cell responses to foreign antigens in now well established (43).

A generally suppressive environment created by Treg cell action would raise the activation threshold for all clonotypes indiscriminately. However, the effect of Treg cells would block the activation of lower avidity clonotypes preferentially, as their threshold may fall below that for effective participation in the response. Indeed, Treg cell activation by IL-2 secreted by the first few effector T cell clonotypes may, in principle, prevent further recruitment of other clonotypes (42). Studies of the CD8^+^ T cell response to *L. monocytogenes* demonstrated that Treg cells inhibit priming selectively of low-avidity CD8^+^ T cell clonotypes, thus improving the overall avidity of the response (44). Studies of the effect of Treg cells on CD4^+^ T cell clonotypes are limited. However, vaccination of a small number of ovarian cancer patients with a peptide from the germ cell protein NY-ESO-1 was shown to induce low-avidity CD4^+^ T cell clonotypes that were insensitive to Treg cell-mediated suppression, suggesting that Treg cells act mostly against high-avidity CD4^+^ T cell clonotypes (45). Although, together these studies highlight the potential of Treg cells to affect the clonotypic composition of an antigen-specific T cell response, additional studies will be required before a consensus emerges.

Another layer of complexity regarding Treg cell-mediated modulation of clonotypic diversity is shared antigen reactivity between Treg cells and effector CD4^+^ T cells. Although high-avidity effector CD4^+^ T cell clonotypes can be efficiently suppressed by Treg cell that do not share antigen reactivity (46), the concomitant presence of Treg cells and effector T cells with the same pMHCII reactivity can often occur (47, 48). It is conceivable that Treg cells have a stronger effect of clonotypic diversity of effector CD4^+^ T cells when their pMHCII reactivity is identical, thus including Treg cells in intra-clonotypic competition.

## Clonotypic Composition According to Antigen Presentation

The overall strength of TCR signal a T cell receives is determined by the TCR affinity for a given pMHCII complex, but it is also affected by the amount or nature of the pMHCII complex itself. Increasing amounts of antigenic pMHCII complexes will prime an increasing number of clonotypes as the activation threshold of lower avidity clonotypes is progressively reached (23, 49). Excessive amounts of antigenic pMHCII complexes or use of higher potency antigenic peptides can lead to the elimination of high-avidity clonotypes, likely through activation-induced cell death (24). Similarly, changes in pMHCII complex as a result of escape mutations in the antigenic peptide will also alter the clonotypic composition of the ensuing T cell response (50). These observations emphasize the potential effect on T cell clonotypic composition of antigen dose and mutability, which in turn affect the relative TCR signal strength each clonotype receives. There are, however, observations where antigen delivery or presentation has been shown to affect the clonotypic composition in ways that are either not fully understood or do not seem to follow simple models of TCR affinity.

### An effect of antigen delivery mode on clonotypic diversity

Early work by Malherbe et al. first demonstrated the powerful effect of the co-administered adjuvant on the overall avidity and clonotypic composition of the CD4^+^ T cell response to immunization with a fixed amount of purified pigeon cytochrome c (PCC) protein (51). The capacity of adjuvants to induce a high-avidity CD4^+^ T cell response was associated with their ability to disperse from the site of injection (51). In addition to different adjuvants in protein immunization, different viral or bacterial vectors used for vaccination of mice against the HIV-1 env were found to induce distinct fine antigen specificities and TCR usage in vaccine-elicited CD8^+^ T cells (52). More recently, the F-MLV env was shown to induce fundamentally different outcomes upon immunization with either the retrovirus or vectors based on recombinant human adenovirus 5 (Ad5) (18). In this study, higher-avidity CD4^+^ T cell responses were linked with faster overall kinetics of the response (18).

Although the choice of adjuvant or vaccine vector can have a profound effect on the clonotypic composition of the elicited T cell response, the underlying mechanisms remain unclear and thus the outcome is not always predictable. Nevertheless, some shared properties of vaccines that induce high-avidity CD4^+^ T cells can be postulated (53). These will undoubtedly include the amount and conformation of antigenic pMHCII complexes produced, the cell type that is presenting them and the inflammatory setting that is generated (53). A common feature of vaccine adjuvants or vectors that elicit higher-avidity CD4^+^ T cell responses seems to be that they also elicit numerically larger responses. Indeed, in the PCC protein immunization system, the adjuvants that induced the highest response magnitude also induced the highest proportion of high-avidity CD4^+^ T cells (51). The same correlation was also observed in the F-MLV env system encoded by either F-MLV or Ad5, and when the vaccine doses were adjusted to ultimately induce a comparable numerical peak in the CD4^+^ T cell response, the response that was faster to reach the peak also contained the highest proportion of high-avidity CD4^+^ T cells (18).

**Figure 2:**
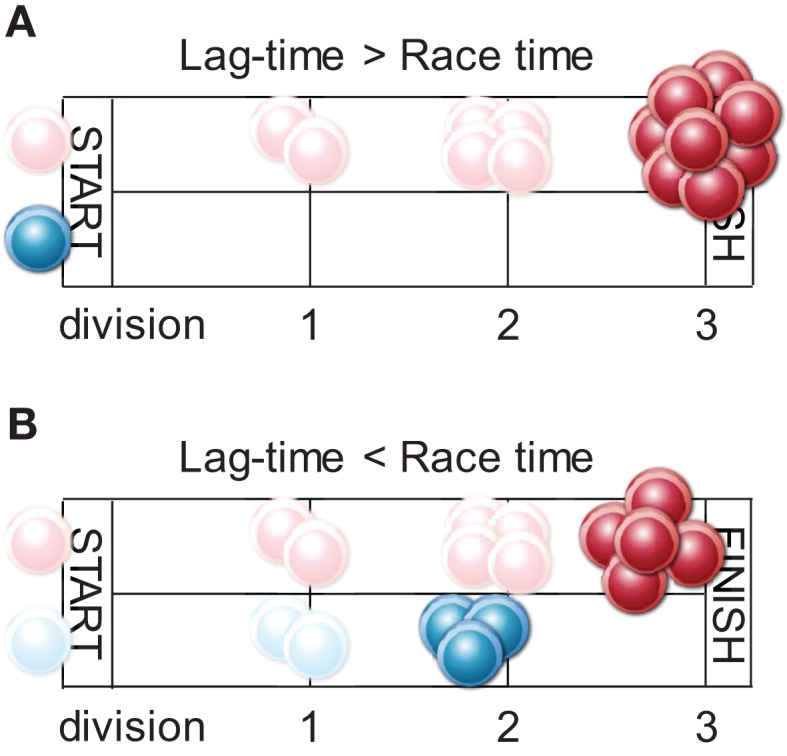
**Antigen-selected repertoire composition according to response kinetics**. The high-affinity clonotype (depicted in red) will begin clonal expansion earlier than the low-affinity one (depicted in blue) as it is more sensitive to initially low antigen concentration during an infection. Clonal expansion will cease when eight antigen-reactive cells are produced (race finish). **(A)** When the lag-time between recruitment of the high- and low-affinity clonotypes is longer than time to expansion of high-affinity clonotype to eight cells, the response will comprise entirely of high-affinity progeny. This may arise when antigenic pMHCII is distributed over many APCs, each presenting at low density. Even though the threshold for activation of the low-affinity clonotype is eventually crossed, its expansion is actively suppressed (race is finished). **(B)** When the lag-time in recruitment is shorter than the time is takes to complete the race (in the case low precursor frequency or when antigen is sharply introduced and highly concentrated on few APCs), the final eight cells will contain a proportion of low-affinity progeny.

If we consider the speed and numerical size of the response as an indicator of the efficiency of antigenic pMHCII presentation, the results of these studies would suggest that fast and strong antigen presentation kinetics result in preferentially high-avidity CD4^+^ T cell responses. This observation would be consistent with a model where the outcome of competition between clonotypes is determined by at least two parameters: first, timing of recruitment, with the higher-avidity clonotypes starting their response earlier; and second; the duration of the response as a whole (Figure 2). The magnitude of the response, counting high- and low-avidity clonotypes collectively, is restricted to a maximum, set by additional T cell-extrinsic parameters. These parameters are not entirely delineated and may include finite availability of growth factors and other factors, collectively described as niche space (54–56). Indeed, comparisons of acute and chronic LCMV or retroviral infection have indicated that virus-specific CD4^+^ T cell numbers achieve comparable magnitudes, following which contraction is initiated, irrespective of viral persistence (57, 58). T cell responses where niche space is filled, and therefore clonal expansion ceases, before low-avidity clonotypes are recruited, will consist entirely of high-avidity clonotypes. In contrast, slower T cell responses, where despite their delayed recruitment, low-avidity clonotypes are allowed time to expand before the total response peaks, will be more diverse (Figure 2). Experimental evidence supporting this notion has been provided in a murine system of CD4^+^ T cell priming to retroviral envelope (18).

### The contribution of APC type to T cell clonotypic composition

Any effect of antigen delivery, production, and presentation will inevitably also depend on the type of APC. The potential of distinct APC types to instruct fundamentally different fates in T cells is perhaps best exemplified by thymic selection, where developing T cells interact with diverse subsets of thymic APCs in discrete thymic microenvironments, resulting in positive and negative selection (59). It is therefore conceivable, that antigen presentation to mature T cells in the periphery may heavily influence the clonotypic composition, according to the particular APC type (53).

Professional APCs, including dendritic cells (DCs), macrophages, and B cells, differ wildly in terms of absolute numbers, anatomical location as well as the way they capture and process exogenous antigen (60–63). In addition to receptors shared with other APCs, B cells can also concentrate limiting amounts of available antigen by virtue of their antigen-specific BCR (64, 65). Nevertheless, antigen can also be captured by non-specific B cells by complement-mediated mechanisms (66–69).

In addition to capturing antigen administered as a vaccine or produced in other cell types, professional APCs may also synthesize antigenic proteins for loading onto MHC II molecules, particularly in the setting of infection. Numerous infectious microbes have been documented to infect professional APCs, including the notable examples of LCMV infection of mouse DCs, Epstein–Barr virus (EBV) infection of human B cells, and *Mycobacterium tuberculosis* infection of mouse and human macrophages (70–72). Importantly, direct infection of professional APCs has, in many cases, been linked to a certain degree of immune subversion or suppression (70–75). Thus, the outcome of presentation by a given APC type may vary depending on whether or not the APC is directly infected.

As well as professional APCs, a variety of hematopoietic and non-hematopoietic cell types are increasingly implicated in MHC II-mediated antigen presentation (76). The overall effect of such atypical APCs on the composition of the CD4^+^ T cell response is not currently understood, but they may favor expansion of unusual clonotypes. Of note, a rhesus cytomegalovirus (RhCMV) vector that was adapted *in vitro* to fibroblast cells and was engineered to express simian immunodeficiency virus (SIV) proteins induced highly unusual SIV-specific CD8^+^ T cells that also reacted with epitopes presented by MHC II molecules (77).

These studies collectively support a role for the type of APC in shaping the clonotypic composition of the CD4^+^ T cell response. However, given the large number of variables potentially dictating the quantity and quality of antigen presentation by different APC types, it is perhaps unsurprising that a clear correlation with the clonotypic composition of the induced T cell response has not yet been made.

## TCR Levels and Signaling Capacity

Besides the affinity of the TCR for pMHCII complexes, the total amount of TCR signaling a CD4^+^ T cell will receive will vary according to the amount of TCR molecules expressed on the T cell surface, as well as the relative capacity of the TCR signaling complex to initiate the signaling cascade. Both of these parameters can change during the course of the immune response and may therefore affect the clonotypic hierarchy.

### Antigen-induced TCR downregulation and clonotypic composition

At steady-state, numbers of surface TCR molecules are maintained at constant levels by balancing production, recycling, and degradation of individual components of the TCR-CD3 signaling complex (78). Upon engagement, surface TCR-CD3 complexes are internalized and recycled or degraded, largely depending on the quality of pMHCII complexes presented by APCs (79, 80). Although antigen-induced TCR internalization may prolong or even amplify TCR signaling from triggered TCR in the immunological synapse, the relative loss of surface TCR reduces new triggering events (79, 80).

The molecular details of TCR downregulation have been elucidated using short *in vitro* assays. However, several infections can cause dramatic downregulation of surface TCR, which can persist for weeks (17, 81–83). In these longer term settings, persistent TCR-CD3 downregulation is thought to render T cells refractory to further antigenic stimulation, thereby contributing to pathogen persistence and the prevention of excessive immune pathology (84).

To what extend TCR downregulation reduces the ability of a T cell to receive further antigenic stimulation, particularly in chronic *in vivo* responses, is not entirely clear. Relative loss of surface TCR levels *per se* may not necessarily compromise TCR signaling capacity. This is due to organization of the surface TCR into oligomers of different sizes prior to antigenic stimulation, which in turn affect sensitivity to antigen (85). Although memory CD4^+^ T cells generally express lower TCR levels than naïve CD4^+^ T cells, TCR signaling is more efficient in memory CD4^+^ T cells as a result of organization of the TCR into larger oligomers in these cells, than in naïve CD4^+^ T cells (86). Moreover, the density of antigenic pMHCII complexes on individual APCs will also modify the degree of TCR downregulation experienced by CD4^+^ T cells, with higher density pMHCII complexes inducing greater loss of surface TCR. Thus, the combined effect of TCR downregulation and TCR oligomerization could either reduce or magnify differences in overall TCR signal strength between clonotypes with high- or low-affinity TCRs.

### TCR signaling settings imprinted prior to antigen encounter

Other than changes in levels and organization of the surface TCR-CD3 complex, accumulating evidence suggests that the TCR signaling capacity in response to antigenic stimulation is modified by the strength of TCR signaling received prior to antigenic encounter. Such signaling originates from recognition of self pMHCII complexes, but may also include environmental antigens from ubiquitous sources or unrelated infections, and has the ability to tune the TCR signal strength (87).

A major function of self pMHCII interaction is to desensitize or eliminate, through negative selection, strongly autoreactive CD4^+^ T cells (87). However, self pMHCII interaction also ensures that CD4^+^ T cells have a fully functional TCR. The latter function is not restricted to passive selection for thymocytes with successfully rearranged TCR; self pMHCII recognition appears to play an active role in setting and maintaining CD4^+^ T cell functionality. Indeed, continuous self pMHCII interaction is necessary for peripheral CD4^+^ T cells to retain full functionality and reactivity with antigenic pMHCII complexes (88, 89). Higher than average affinity for self pMHCII complexes during successful selection of thymocytes establishes in mature T cells a modified naïve phenotype characterized by elevated expression of CD5 and reduced expression of Ly6C (90, 91). Recent studies have highlighted the importance of self pMHCII interaction during thymocyte development and later in the periphery in determining the relative success of mature CD4^+^ and CD8^+^ T clonotypes during subsequent responses to antigenic stimulation (92–94).

The precise relationship between affinity for self or antigenic pMHCII complexes and clonal expansion in response to antigen is not fully delineated and may depend on additional parameters. In a simple model, clonotypes with the strongest reactivity with self pMHCII complexes will also display the strongest reactivity with antigenic pMHCII complexes, likely as a result of stronger TCR binding to the latter complexes (93). Alternatively, clonotypes with stronger reactivity with self pMHCII complexes will react stronger with antigenic pMHCII, not due to increased affinity of the selected TCR for antigenic pMHCII, but due to more efficient TCR signaling in these cells (92). Moreover, reactivity with self pMHCII complexes does not always predict which clonotype will dominate the CD4^+^ T cell response (82, 92, 93).

The proposed dominant effect of self-reactivity on CD4^+^ T cell responsiveness to antigenic stimulation (92, 93) seemingly contrasts the evidence supporting a role for affinity to antigenic pMHCII driving clonotypic competition. The relative importance of either self or antigenic pMHCII complexes in determining the clonotypic composition of the CD4^+^ T cell response will depend on multiple factors. These will include the relative abundance of self and antigenic pMHCII complexes at various stages of the response. For example, whereas self pMHCII complexes shape T cell repertoires and TCR responsiveness already in the thymus and they are assumed constitutively present in the periphery, antigenic pMHCII complexes will exhibit dramatic changes in abundance in the course of an infection. At the peak of infection, the effect of antigenic pMHCII complexes may be stronger and overshadow that of self pMHCII complexes. In contrast, as infection is controlled and antigenic pMHCII complexes become limiting, the role of self pMHCII complexes may become increasingly dominant.

**Figure 3:**
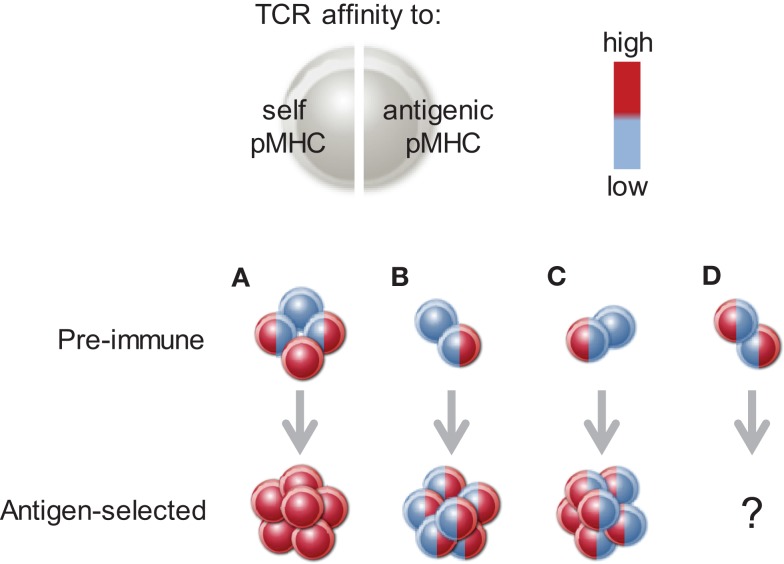
**The balance of reactivity to self or antigen pMHCII complexes**. The TCR of a given T cell may have high (red) or low (blue) affinity for self pMHCII (left side of the cell) or a particular antigenic pMHCII complex (right side of the cell). High affinity to either of these contributes positively to clonal expansion. **(A)** When all four possible (simplified) combinations are present, the clonotype that combines high affinity for self and for antigenic pMHCII will prevail. Selection of such clonotypes from large enough repertoires may create the impression that high affinity to self and antigen are positively correlated. **(B)** Between two clonotypes with equal affinity to self, the one with higher affinity to antigen will prevail. **(C)** Between two clonotypes with equal affinity to antigen, the one with higher affinity to self will prevail. **(D)** The outcome of competition between clonotypes one of which is high affinity for self and the other for antigen is currently difficult to predict.

In the same way, relative importance of either self or antigenic pMHCII complexes will also depend on the affinity of individual clonotypes for either type of TCR stimulation (Figure 3). When the affinity of two distinct clonotypes for the same antigenic pMHCII complex is comparable, the difference in clonal expansion will derive from differences in self-reactivity, thus emphasizing the contribution of the latter. Conversely, when self-reactivity of two distinct clonotypes is comparable, then differences in clonal expansion will depend on affinity for antigenic pMHCII complexes. In a polyclonal repertoire, where clonotypes with equally high affinity for a foreign antigen can be drawn from CD4^+^ T cell pools with either low or high self-reactivity, the fraction with higher self-reactivity may win the competition. The use of CD5 as a marker for self-reactivity has provided strong evidence that self-reactivity optimizes reactivity to foreign antigen (93). However, if as a result of self-tolerance, the fraction with high self-reactivity contained clonotypes with only low affinity for a particular antigenic pMHCII complex, then clonotypes with high affinity for that antigen in the fraction with low self-reactivity may win the competition (Figure 3). Antigens with significant similarity to self proteins may fall in the second category. Indeed, the presence of endogenous retroviruses in the germ-line has been shown to create partial tolerance to exogenous retroviral proteins, resulting in a repertoire where clonotypes with higher reactivity with endogenous retroviral antigens display lower reactivity with exogenous antigen and are outcompeted during retroviral infection (95).

## TCR Signals during Priming Affecting T Cell Maintenance

An overwhelming amount of data supports a role for strong TCR signaling in CD4^+^ T cell expansion and differentiation during priming. Continuous TCR signals also contribute to successful CD4^+^ T cell memory formation (22, 96–98) and homeostatic competition between memory CD4^+^ T cell clonotypes (99). However, settings can also be considered where the same strong TCR signals that promote CD4^+^ T cell expansion during the early phases of the response compromise the ability of T cells to maintain their presence. In analogy to the antagonistic pleiotropy hypothesis, first proposed to explain how a given gene product can increase fitness early in life, but cause aging later in life (100), what drives early fitness of a particular CD4^+^ T cell clonotype may lead to its demise later in the response.

### Negative feedback pathways tuning the T cell response

Central to the ability of the host to mount a strong T cell response is also the ability to regulate potential over-reactivity and many cell-autonomous layers of negative regulators have been described. The most extreme form of negative regulation is death of T cells as a direct result of strong or persistent TCR signaling (101), a mechanism that has been incriminated in the loss of high-affinity CD4^+^ T cell clonotypes following immunization with high potency agonists (24). However, there are less extreme negative feedback pathways that may not lead to the complete elimination of the highest affinity clonotypes, but rather curtail their dominance. CTLA-4 can regulate T cell responses both cell-intrinsically and -extrinsically (40, 102). Notably, CTLA-4 accumulation at the immunological synapse is proportional to the strength of TCR signaling and, by extension, to TCR affinity (103). As a result, cell-intrinsic T cell inhibition by CTLA-4 is expected to affect higher affinity CD4^+^ T cell clonotypes more than lower-affinity ones. Similarly, strong TCR activation also induces PD-1 expression, which in turn inhibits TCR signaling (104). Thus, a direct link between TCR signal strength and expression of inhibitory receptors such as CTLA-4 and PD-1 can be considered as a negative feedback mechanism preserving clonotypic diversity by limiting the expansion of higher affinity CD4^+^ T cell clonotypes.

### Clonotypic composition according to clonotypic Th subset differentiation

TCR signal strength can heavily skew Th differentiation to or away from particular Th subsets (13). Strong and weak TCR signals are generally inducing Th1 and Th2 differentiation, respectively (105–108). Strong TCR signals can also promote T follicular helper (Tfh) cells in certain responses (9, 109), although efficient Tfh differentiation of clonotypes receiving weak TCR stimulation has also been observed in other responses (17, 110, 111). Moreover, clonotypes with higher self-reactivity show a bias in Treg cell conversion (90).

The relative ability of different Th subsets to form a stable memory population has not been systematically compared and may vary according to the infection or immunization. For example, maintenance of Tfh cells is thought to require persistent antigen and germinal center B cells (112), and consequently Tfh cell numbers would be expected to decline when the germinal center reaction ceases. Nevertheless, Tfh cell numbers have also been reported to increase in the chronic phase of LCMV infection (113, 114) or to stably persist as memory in other settings (115–118). Th1, Th2, Th17, and Treg cells can also display variable kinetics, stability, or plasticity depending on infection and antigen parameters (54, 119–122).

Thus, CD4^+^ T cell clonotypes will, to a certain degree, assort into distinct Th functional subsets, each of which may exhibit differential capacity to persist into memory, in turn shifting the clonotypic composition of the CD4^+^ T cell response over time. For instance, a low-affinity clonotype may be preferentially enriched in a chronic response due to its skewed differentiation into a Th subset with increased numerical stability.

The relative stability of Th subsets, and by extension of the clonotypes that preferentially differentiate into those subsets, can additionally be affected by extrinsic infection-related factors. CD4^+^ T cells clonotypes with the highest affinity for antigenic pMHCII complexes will outcompete other lower-affinity clonotypes, but may be lost due to preferential infection in the case of T cell-tropic viruses (123, 124). Similarly, high-affinity CD4^+^ T cell clonotypes skewed to Th1 differentiation will preferentially migrate to the inflamed tissue in the case of granulomatous bacterial infections, where they are more likely to die than clonotypes that do not show this behavior (125).

## Concluding Remarks

The energetically costly generation of a diverse TCR and BCR repertoire underpins the evolutionary success of adaptive immunity as it is critical for its function (5, 126). The obvious advantage of TCR repertoire diversity is to allow the selection of at least one T cell clonotype, best fit to respond to a particular antigen. Clonal expansion of such a T cell clonotype ensures effective immunity and contributes to immunological memory. However, the potential advantage of TCR repertoire diversity in the response to a particular antigen may be more difficult to quantify. In principle, sufficient numbers of a single T cell clonotype with the optimal TCR affinity could competently provide immune protection. However, there may be distinct advantages of preserving diversity in the antigen-selected TCR repertoire.

Although a single naïve T cell can give progeny that differentiates into multiple functional subsets (9, 127), distinct CD4^+^ T cell clonotypes will generate different Th subset ratios (9). Thus, the maximum Th subset diversity can only be achieved by sufficiently large TCR diversity.

As the number of available TCRs is finite and relatively small in comparison to the number of potential antigens, TCR cross-reactivity is essential to broaden immune coverage by the naïve repertoire (5). This property will remain essential in the antigen-selected TCR repertoire. As different TCR that share reactivity with a given antigen display varying amounts of cross-reactivity with altered variants of this antigen, TCR diversity in the antigen-specific response will expand the number of antigenic variants that can be recognized by antigen-selected T cells. This will be particularly important in infections where the speed of escape mutations present a challenge to the adaptability of the immune response (128).

Another potential advantage of diversity in the CD4^+^ T cell response to a particular antigen relates to the clonal nature of TCR distribution. Even if sufficient for protective immunity, a single clonal T cell family may inherit the characteristics of the single thymic selection event imprinted onto the founder cell of that family. A polyclonal response will comprise clones from separate thymic selection events, life-histories and likely different patterns of TCR responsiveness, self-reactivity, and survival potential.

The emerging picture is of numerous factors that maximize diversity in the CD4^+^ T cell response, by alleviating any disadvantage of lower-affinity clonotypes. Some of these factors are extrinsic to T cells and can be manipulated in our attempts to induce protective CD4^+^ T cell immunity or prevent autoimmunity. However, any intervention toward TCR repertoire “engineering” will necessitate deeper understanding of the type of TCR diversity that best fits a particular response, and this should be the focus of future investigation.

## Conflict of Interest Statement

The authors declare that the research was conducted in the absence of any commercial or financial relationships that could be construed as a potential conflict of interest.

## References

[1] Davis MM Bjorkman PJ. T-cell antigen receptor genes and T-cell recognition. Nature (1988) 334:395–402. 10.1038/334395a0 3043226

[2] Murugan A Mora T Walczak AM Callan CG Jr. Statistical inference of the generation probability of T-cell receptors from sequence repertoires. Proc Natl Acad Sci U S A (2012) 109:16161–6. 10.1073/pnas.1212755109 22988065 PMC3479580

[3] Robins HS Srivastava SK Campregher PV Turtle CJ Andriesen J Riddell SR Overlap and effective size of the human CD8+ T cell receptor repertoire. Sci Transl Med (2010) 2:47ra64. 10.1126/scitranslmed.3001442 20811043 PMC3212437

[4] Zarnitsyna VI Evavold BD Schoettle LN Blattman JN Antia R. Estimating the diversity, completeness, and cross-reactivity of the T cell repertoire. Front Immunol (2013) 4:485. 10.3389/fimmu.2013.00485 24421780 PMC3872652

[5] Nikolich-Zugich J Slifka MK Messaoudi I. The many important facets of T-cell repertoire diversity. Nat Rev Immunol (2004) 4:123–32. 10.1038/nri1292 15040585

[6] Casrouge A Beaudoing E Dalle S Pannetier C Kanellopoulos J Kourilsky P. Size estimate of the alpha beta TCR repertoire of naive mouse splenocytes. J Immunol (2000) 164:5782–7. 10.4049/jimmunol.164.11.5782 10820256

[7] Moon JJ Chu HH Pepper M McSorley SJ Jameson SC Kedl RM Naive CD4(+) T cell frequency varies for different epitopes and predicts repertoire diversity and response magnitude. Immunity (2007) 27:203–13. 10.1016/j.immuni.2007.07.007 17707129 PMC2200089

[8] Jenkins MK Chu HH McLachlan JB Moon JJ. On the composition of the preimmune repertoire of T cells specific for peptide-major histocompatibility complex ligands. Annu Rev Immunol (2010) 28:275–94. 10.1146/annurev-immunol-030409-101253 20307209

[9] Tubo NJ Pagan AJ Taylor JJ Nelson RW Linehan JL Ertelt JM Single naive CD4(+) T cells from a diverse repertoire produce different effector cell types during infection. Cell (2013) 153:785–96. 10.1016/j.cell.2013.04.007 23663778 PMC3766899

[10] Corse E Gottschalk RA Allison JP. Strength of TCR-peptide/MHC interactions and in vivo T cell responses. J Immunol (2011) 186:5039–45. 10.4049/jimmunol.1003650 21505216

[11] Stone JD Chervin AS Kranz DM. T-cell receptor binding affinities and kinetics: impact on T-cell activity and specificity. Immunology (2009) 126:165–76. 10.1111/j.1365-2567.2008.03015.x 19125887 PMC2632691

[12] Thorborn G Young GR Kassiotis G. Effective T helper cell responses against retroviruses: are all clonotypes equal? J Leukoc Biol (2014) 96:27–37. 10.1189/jlb.2RI0613-347R 24737804

[13] Tubo NJ Jenkins MK. TCR signal quantity and quality in CD4 T cell differentiation. Trends Immunol (2014) 35:591–6. 10.1016/j.it.2014.09.008 25457838 PMC4406772

[14] Fasso M Anandasabapathy N Crawford F Kappler J Fathman CG Ridgway WM. T cell receptor (TCR)-mediated repertoire selection and loss of TCR vbeta diversity during the initiation of a CD4(+) T cell response in vivo. J Exp Med (2000) 192:1719–30. 10.1084/jem.192.12.1719 11120769 PMC2213496

[15] Gett AV Sallusto F Lanzavecchia A Geginat J. T cell fitness determined by signal strength. Nat Immunol (2003) 4:355–60. 10.1038/ni908 12640450

[16] Malherbe L Hausl C Teyton L McHeyzer-Williams MG. Clonal selection of helper T cells is determined by an affinity threshold with no further skewing of TCR binding properties. Immunity (2004) 21:669–79. 10.1016/j.immuni.2004.09.008 15539153

[17] Ploquin MJ Eksmond U Kassiotis G. B cells and TCR avidity determine distinct functions of CD4+ T cells in retroviral infection. J Immunol (2011) 187:3321–30. 10.4049/jimmunol.1101006 21841129 PMC3173872

[18] Thorborn G Ploquin MJ Eksmond U Pike R Bayer W Dittmer U Clonotypic composition of the CD4+ T cell response to a vectored retroviral antigen is determined by its speed. J Immunol (2014) 193:1567–77. 10.4049/jimmunol.1400667 25000983 PMC4119786

[19] McHeyzer-Williams MG Davis MM. Antigen-specific development of primary and memory T cells in vivo. Science (1995) 268:106–11. 10.1126/science.7535476 7535476

[20] Savage PA Boniface JJ Davis MM. A kinetic basis for T cell receptor repertoire selection during an immune response. Immunity (1999) 10:485–92. 10.1016/S1074-7613(00)80048-5 10229191

[21] McHeyzer-Williams LJ Panus JF Mikszta JA McHeyzer-Williams MG. Evolution of antigen-specific T cell receptors in vivo: preimmune and antigen-driven selection of preferred complementarity-determining region 3 (CDR3) motifs. J Exp Med (1999) 189:1823–38. 10.1084/jem.189.11.1823 10359586 PMC2193074

[22] Kim C Wilson T Fischer KF Williams MA. Sustained interactions between T cell receptors and antigens promote the differentiation of CD4(+) memory T cells. Immunity (2013) 39:508–20. 10.1016/j.immuni.2013.08.033 24054329 PMC3816772

[23] Rees W Bender J Teague TK Kedl RM Crawford F Marrack P An inverse relationship between T cell receptor affinity and antigen dose during CD4(+) T cell responses in vivo and in vitro. Proc Natl Acad Sci U S A (1999) 96:9781–6. 10.1073/pnas.96.17.9781 10449771 PMC22287

[24] Anderton SM Radu CG Lowrey PA Ward ES Wraith DC. Negative selection during the peripheral immune response to antigen. J Exp Med (2001) 193:1–11. 10.1084/jem.193.1.1 11136816 PMC2195878

[25] Caserta S Kleczkowska J Mondino A Zamoyska R. Reduced functional avidity promotes central and effector memory CD4 T cell responses to tumor-associated antigens. J Immunol (2010) 185:6545–54. 10.4049/jimmunol.1001867 21048115

[26] Ertelt JM Johanns TM Mysz MA Nanton MR Rowe JH Aguilera MN Selective culling of high avidity antigen-specific CD4+ T cells after virulent *Salmonella* infection. Immunology (2011) 134:487–97. 10.1111/j.1365-2567.2011.03510.x 22044420 PMC3230801

[27] Baumgartner CK Yagita H Malherbe LP. A TCR affinity threshold regulates memory CD4 T cell differentiation following vaccination. J Immunol (2012) 189:2309–17. 10.4049/jimmunol.1200453 22844120 PMC3424363

[28] Foulds KE Shen H. Clonal competition inhibits the proliferation and differentiation of adoptively transferred TCR transgenic CD4 T cells in response to infection. J Immunol (2006) 176:3037–43. 10.4049/jimmunol.176.5.3037 16493062

[29] Weaver JM Chaves FA Sant AJ. Abortive activation of CD4 T cell responses during competitive priming in vivo. Proc Natl Acad Sci U S A (2009) 106:8647–52. 10.1073/pnas.0811584106 19423666 PMC2689016

[30] Whitmire JK Benning N Eam B Whitton JL. Increasing the CD4+ T cell precursor frequency leads to competition for IFN-gamma thereby degrading memory cell quantity and quality. J Immunol (2008) 180:6777–85. 10.4049/jimmunol.180.10.6777 18453598 PMC2788825

[31] Blair DA Lefrancois L. Increased competition for antigen during priming negatively impacts the generation of memory CD4 T cells. Proc Natl Acad Sci U S A (2007) 104:15045–50. 10.1073/pnas.0703767104 17827281 PMC1986610

[32] Williams MA Ravkov EV Bevan MJ. Rapid culling of the CD4+ T cell repertoire in the transition from effector to memory. Immunity (2008) 28:533–45. 10.1016/j.immuni.2008.02.014 18356084 PMC2391296

[33] Watts TH. TNF/TNFR family members in costimulation of T cell responses. Annu Rev Immunol (2005) 23:23–68. 10.1146/annurev.immunol.23.021704.115839 15771565

[34] Croft M. Co-stimulatory members of the TNFR family: keys to effective T-cell immunity? Nat Rev Immunol (2003) 3:609–20. 10.1038/nri1148 12974476

[35] Greenwald RJ Freeman GJ Sharpe AH The B7 family revisited. Annu Rev Immunol (2005) 23:515–48. 10.1146/annurev.immunol.23.021704.115611 15771580

[36] van Gisbergen KP Klarenbeek PL Kragten NA Unger PP Nieuwenhuis MB Wensveen FM The costimulatory molecule CD27 maintains clonally diverse CD8(+) T cell responses of low antigen affinity to protect against viral variants. Immunity (2011) 35:97–108. 10.1016/j.immuni.2011.04.020 21763160

[37] Pepper M Linehan JL Pagan AJ Zell T Dileepan T Cleary PP Different routes of bacterial infection induce long-lived TH1 memory cells and short-lived TH17 cells. Nat Immunol (2010) 11:83–9. 10.1038/ni.1826 19935657 PMC2795784

[38] Hodge JW Chakraborty M Kudo-Saito C Garnett CT Schlom J. Multiple costimulatory modalities enhance CTL avidity. J Immunol (2005) 174:5994–6004. 10.4049/jimmunol.174.12.8220 15879092 PMC1924685

[39] Kuhns MS Epshteyn V Sobel RA Allison JP. Cytotoxic T lymphocyte antigen-4 (CTLA-4) regulates the size, reactivity, and function of a primed pool of CD4+ T cells. Proc Natl Acad Sci U S A (2000) 97:12711–6. 10.1073/pnas.220423597 11050166 PMC18829

[40] Qureshi OS Zheng Y Nakamura K Attridge K Manzotti C Schmidt EM Trans-endocytosis of CD80 and CD86: a molecular basis for the cell-extrinsic function of CTLA-4. Science (2011) 332:600–3. 10.1126/science.1202947 21474713 PMC3198051

[41] Callard RE Stark J Yates AJ. Fratricide: a mechanism for T memory-cell homeostasis. Trends Immunol (2003) 24:370–5. 10.1016/S1471-4906(03)00164-9 12860527

[42] Almeida AR Amado IF Reynolds J Berges J Lythe G Molina-Paris C Quorum-sensing in CD4(+) T cell homeostasis: a hypothesis and a model. Front Immunol (2012) 3:125. 10.3389/fimmu.2012.00125 22654881 PMC3360200

[43] Josefowicz SZ Lu LF Rudensky AY. Regulatory T cells: mechanisms of differentiation and function. Annu Rev Immunol (2012) 30:531–64. 10.1146/annurev.immunol.25.022106.141623 22224781 PMC6066374

[44] Pace L Tempez A Arnold-Schrauf C Lemaitre F Bousso P Fetler L Regulatory T cells increase the avidity of primary CD8+ T cell responses and promote memory. Science (2012) 338:532–6. 10.1126/science.1227049 23112334

[45] Nishikawa H Qian F Tsuji T Ritter G Old LJ Gnjatic S Influence of CD4+CD25+ regulatory T cells on low/high-avidity CD4+ T cells following peptide vaccination. J Immunol (2006) 176:6340–6. 10.4049/jimmunol.176/10/6340 16670346

[46] Antunes I Tolaini M Kissenpfennig A Iwashiro M Kuribayashi K Malissen B Retrovirus-specificity of regulatory T cells is neither present nor required in preventing retrovirus-induced bone marrow immune pathology. Immunity (2008) 29:782–94. 10.1016/j.immuni.2008.09.016 19006695 PMC2631611

[47] Veiga-Parga T Sehrawat S Rouse BT. Role of regulatory T cells during virus infection. Immunol Rev (2013) 255:182–96. 10.1111/imr.12085 23947355 PMC3748387

[48] Kretschmer K Apostolou I Jaeckel E Khazaie K von BH. Making regulatory T cells with defined antigen specificity: role in autoimmunity and cancer. Immunol Rev (2006) 212:163–9. 10.1111/j.0105-2896.2006.00411.x 16903913

[49] Vanguri V Govern CC Smith R Huseby ES. Viral antigen density and confinement time regulate the reactivity pattern of CD4 T-cell responses to vaccinia virus infection. Proc Natl Acad Sci U S A (2013) 110:288–93. 10.1073/pnas.1208328110 23248307 PMC3538251

[50] McMichael AJ Phillips RE. Escape of human immunodeficiency virus from immune control. Annu Rev Immunol (1997) 15:271–96. 10.1146/annurev.immunol.15.1.271 9143689

[51] Malherbe L Mark L Fazilleau N McHeyzer-Williams LJ McHeyzer-Williams MG. Vaccine adjuvants alter TCR-based selection thresholds. Immunity (2008) 28:698–709. 10.1016/j.immuni.2008.03.014 18450485 PMC2695494

[52] Honda M Wang R Kong WP Kanekiyo M Akahata W Xu L Different vaccine vectors delivering the same antigen elicit CD8+ T cell responses with distinct clonotype and epitope specificity. J Immunol (2009) 183:2425–34. 10.4049/jimmunol.0900581 19620307 PMC2858449

[53] Baumgartner CK Malherbe LP. Regulation of CD4 T-cell receptor diversity by vaccine adjuvants. Immunology (2010) 130:16–22. 10.1111/j.1365-2567.2010.03265.x 20331477 PMC2855789

[54] Stockinger B Kassiotis G Bourgeois C. CD4 T-cell memory. Semin Immunol (2004) 16:295–303. 10.1016/j.smim.2004.08.010 15528074

[55] Taylor JJ Jenkins MK. CD4+ memory T cell survival. Curr Opin Immunol (2011) 23:319–23. 10.1016/j.coi.2011.03.010 21524898

[56] Welsh RM Selin LK Szomolanyi-Tsuda E Immunological memory to viral infections. Annu Rev Immunol (2004) 22:711–43. 10.1146/annurev.immunol.22.012703.104527 15032594

[57] Pike R Filby A Ploquin MJ Eksmond U Marques R Antunes I Race between retroviral spread and CD4+ T-cell response determines the outcome of acute friend virus infection. J Virol (2009) 83:11211–22. 10.1128/JVI.01225-09 19692462 PMC2772778

[58] Brooks DG Teyton L Oldstone MB McGavern DB. Intrinsic functional dysregulation of CD4 T cells occurs rapidly following persistent viral infection. J Virol (2005) 79:10514–27. 10.1128/JVI.79.16.10514-10527.2005 16051844 PMC1182641

[59] Klein L Kyewski B Allen PM Hogquist KA. Positive and negative selection of the T cell repertoire: what thymocytes see (and don’t see). Nat Rev Immunol (2014) 14:377–91. 10.1038/nri3667 24830344 PMC4757912

[60] Watts C. The exogenous pathway for antigen presentation on major histocompatibility complex class II and CD1 molecules. Nat Immunol (2004) 5:685–92. 10.1038/ni1088 15224094

[61] Manoury B. Proteases: essential actors in processing antigens and intracellular toll-like receptors. Front Immunol (2013) 4:299. 10.3389/fimmu.2013.00299 24065969 PMC3781364

[62] Banchereau J Briere F Caux C Davoust J Lebecque S Liu YJ Immunobiology of dendritic cells. Annu Rev Immunol (2000) 18:767–811. 10.1146/annurev.immunol.18.1.767 10837075

[63] Trombetta ES Mellman I. Cell biology of antigen processing in vitro and in vivo. Annu Rev Immunol (2005) 23:975–1028. 10.1146/annurev.immunol.22.012703.104538 15771591

[64] Lanzavecchia A. Antigen-specific interaction between T and B cells. Nature (1985) 314:537–9. 10.1038/314537a0 3157869

[65] Yuseff MI Pierobon P Reversat A Lennon-Dumenil AM. How B cells capture, process and present antigens: a crucial role for cell polarity. Nat Rev Immunol (2013) 13:475–86. 10.1038/nri3469 23797063

[66] Bila C Oberhauser V Ammann CG Ejaz A Huber G Schimmer S Complement opsonization enhances friend virus infection of B cells and thereby amplifies the virus-specific CD8+ T cell response. J Virol (2011) 85:1151–5. 10.1128/JVI.01821-10 21047954 PMC3019994

[67] Carroll MC. The role of complement and complement receptors in induction and regulation of immunity. Annu Rev Immunol (1998) 16:545–68. 10.1146/annurev.immunol.16.1.545 9597141

[68] Barrault DV Knight AM. Distinct sequences in the cytoplasmic domain of complement receptor 2 are involved in antigen internalization and presentation. J Immunol (2004) 172:3509–17. 10.4049/jimmunol.172.6.3509 15004151

[69] Nielsen CH Leslie RG Jepsen BS Kazatchkine MD Kaveri SV Fischer E. Natural autoantibodies and complement promote the uptake of a self antigen, human thyroglobulin, by B cells and the proliferation of thyroglobulin-reactive CD4(+) T cells in healthy individuals. Eur J Immunol (2001) 31:2660–8. 10.1002/1521-4141(200109)31 11536164

[70] Sevilla N Kunz S McGavern D Oldstone MB. Infection of dendritic cells by lymphocytic choriomeningitis virus. Curr Top Microbiol Immunol (2003) 276:125–44. 10.1007/978-3-662-06508-2_6 12797446 PMC5321679

[71] Hatton OL Harris-Arnold A Schaffert S Krams SM Martinez OM. The interplay between Epstein-Barr virus and B lymphocytes: implications for infection, immunity, and disease. Immunol Res (2014) 58:268–76. 10.1007/s12026-014-8496-1 24619311 PMC4199828

[72] Hmama Z Pena-Diaz S Joseph S Av-Gay Y. Immunoevasion and immunosuppression of the macrophage by *Mycobacterium tuberculosis*. Immunol Rev (2015) 264:220–32. 10.1111/imr.12268 25703562

[73] Nothelfer K Sansonetti PJ Phalipon A. Pathogen manipulation of B cells: the best defence is a good offence. Nat Rev Microbiol (2015) 13:173–84. 10.1038/nrmicro3415 25659322

[74] van KY Geijtenbeek TB. DC-SIGN: escape mechanism for pathogens. Nat Rev Immunol (2003) 3:697–709. 10.1038/nri1182 12949494

[75] Harding CV Boom WH. Regulation of antigen presentation by *Mycobacterium tuberculosis*: a role for toll-like receptors. Nat Rev Microbiol (2010) 8:296–307. 10.1038/nrmicro2321 20234378 PMC3037727

[76] Kambayashi T Laufer TM. Atypical MHC class II-expressing antigen-presenting cells: can anything replace a dendritic cell? Nat Rev Immunol (2014) 14:719–30. 10.1038/nri3754 25324123

[77] Hansen SG Sacha JB Hughes CM Ford JC Burwitz BJ Scholz I *Cytomegalovirus* vectors violate CD8+ T cell epitope recognition paradigms. Science (2013) 340:1237874. 10.1126/science.1237874 23704576 PMC3816976

[78] Alcover A Alarcon B. Internalization and intracellular fate of TCR-CD3 complexes. Crit Rev Immunol (2000) 20:325–46. 10.1615/CritRevImmunol.v20.i4.20 11100805

[79] Valitutti S Muller S Cella M Padovan E Lanzavecchia A. Serial triggering of many T-cell receptors by a few peptide-MHC complexes. Nature (1995) 375:148–51. 10.1038/375148a0 7753171

[80] Lanzavecchia A Sallusto F. Antigen decoding by T lymphocytes: from synapses to fate determination. Nat Immunol (2001) 2:487–92. 10.1038/88678 11376334

[81] Gallimore A Glithero A Godkin A Tissot AC Pluckthun A Elliott T Induction and exhaustion of lymphocytic choriomeningitis virus-specific cytotoxic T lymphocytes visualized using soluble tetrameric major histocompatibility complex class I-peptide complexes. J Exp Med (1998) 187:1383–93. 10.1084/jem.187.9.1383 9565631 PMC2212278

[82] Weber KS Li QJ Persaud SP Campbell JD Davis MM Allen PM. Distinct CD4+ helper T cells involved in primary and secondary responses to infection. Proc Natl Acad Sci U S A (2012) 109:9511–6. 10.1073/pnas.1202408109 22645349 PMC3386110

[83] Munitic I Decaluwe H Evaristo C Lemos S Wlodarczyk M Worth A Epitope specificity and relative clonal abundance do not affect CD8 differentiation patterns during lymphocytic choriomeningitis virus infection. J Virol (2009) 83:11795–807. 10.1128/JVI.01402-09 19726518 PMC2772677

[84] Baniyash M. TCR zeta-chain downregulation: curtailing an excessive inflammatory immune response. Nat Rev Immunol (2004) 4:675–87. 10.1038/nri1434 15343367

[85] Schamel WW Arechaga I Risueno RM van Santen HM Cabezas P Risco C Coexistence of multivalent and monovalent TCRs explains high sensitivity and wide range of response. J Exp Med (2005) 202:493–503. 10.1084/jem.20042155 16087711 PMC2212847

[86] Kumar R Ferez M Swamy M Arechaga I Rejas MT Valpuesta JM Increased sensitivity of antigen-experienced T cells through the enrichment of oligomeric T cell receptor complexes. Immunity (2011) 35:375–87. 10.1016/j.immuni.2011.08.010 21903423

[87] Hogquist KA Jameson SC. The self-obsession of T cells: how TCR signaling thresholds affect fate ‘decisions’ and effector function. Nat Immunol (2014) 15:815–23. 10.1038/ni.2938 25137456 PMC4348363

[88] Kassiotis G Garcia S Simpson E Stockinger B. Impairment of immunological memory in the absence of MHC despite survival of memory T cells. Nat Immunol (2002) 3:244–50. 10.1038/ni766 11836529

[89] Stefanova I Dorfman JR Germain RN. Self-recognition promotes the foreign antigen sensitivity of naive T lymphocytes. Nature (2002) 420:429–34. 10.1038/nature01146 12459785

[90] Martin B Auffray C Delpoux A Pommier A Durand A Charvet C Highly self-reactive naive CD4 T cells are prone to differentiate into regulatory T cells. Nat Commun (2013) 4:2209. 10.1038/ncomms3209 23900386

[91] Azzam HS Grinberg A Lui K Shen H Shores EW Love PE. CD5 expression is developmentally regulated by T cell receptor (TCR) signals and TCR avidity. J Exp Med (1998) 188:2301–11. 10.1084/jem.188.12.2301 9858516 PMC2212429

[92] Persaud SP Parker CR Lo WL Weber KS Allen PM. Intrinsic CD4(+) T cell sensitivity and response to a pathogen are set and sustained by avidity for thymic and peripheral complexes of self peptide and MHC. Nat Immunol (2014) 15:266–74. 10.1038/ni.2822 24487322 PMC3944141

[93] Mandl JN Monteiro JP Vrisekoop N Germain RN. T cell-positive selection uses self-ligand binding strength to optimize repertoire recognition of foreign antigens. Immunity (2013) 38:263–74. 10.1016/j.immuni.2012.09.011 23290521 PMC3785078

[94] Fulton RB Hamilton SE Xing Y Best JA Goldrath AW Hogquist KA The TCR’s sensitivity to self peptide-MHC dictates the ability of naive CD8(+) T cells to respond to foreign antigens. Nat Immunol (2015) 16:107–17. 10.1038/ni.3043 25419629 PMC4270846

[95] Young GR Ploquin MJ Eksmond U Wadwa M Stoye JP Kassiotis G. Negative selection by an endogenous retrovirus promotes a higher-avidity CD4+ T cell response to retroviral infection. PLoS Pathog (2012) 8:e1002709. 10.1371/journal.ppat.1002709 22589728 PMC3349761

[96] Obst R van Santen HM Mathis D Benoist C. Antigen persistence is required throughout the expansion phase of a CD4(+) T cell response. J Exp Med (2005) 201:1555–65. 10.1084/jem.20042521 15897273 PMC2212918

[97] Celli S Lemaitre F Bousso P. Real-time manipulation of T cell-dendritic cell interactions in vivo reveals the importance of prolonged contacts for CD4+ T cell activation. Immunity (2007) 27:625–34. 10.1016/j.immuni.2007.08.018 17950004

[98] Yarke CA Dalheimer SL Zhang N Catron DM Jenkins MK Mueller DL. Proliferating CD4+ T cells undergo immediate growth arrest upon cessation of TCR signaling in vivo. J Immunol (2008) 180:156–62. 10.4049/jimmunol.180.1.156 18097015

[99] Kassiotis G Zamoyska R Stockinger B. Involvement of avidity for major histocompatibility complex in homeostasis of naive and memory T cells. J Exp Med (2003) 197:1007–16. 10.1084/jem.20021812 12707300 PMC2193871

[100] Williams G Pleiotropy C Natural selection, and the evolution of senescence. Evolution (1957) 11:398–411. 10.2307/2406060

[101] Krammer PH Arnold R Lavrik IN. Life and death in peripheral T cells. Nat Rev Immunol (2007) 7:532–42. 10.1038/nri2115 17589543

[102] Wing K Yamaguchi T Sakaguchi S. Cell-autonomous and -non-autonomous roles of CTLA-4 in immune regulation. Trends Immunol (2011) 32:428–33. 10.1016/j.it.2011.06.002 21723783

[103] Egen JG Allison JP. Cytotoxic T lymphocyte antigen-4 accumulation in the immunological synapse is regulated by TCR signal strength. Immunity (2002) 16:23–35. 10.1016/S1074-7613(01)00259-X 11825563

[104] Okazaki T Chikuma S Iwai Y Fagarasan S Honjo T. A rheostat for immune responses: the unique properties of PD-1 and their advantages for clinical application. Nat Immunol (2013) 14:1212–8. 10.1038/ni.2762 24240160

[105] Hosken NA Shibuya K Heath AW Murphy KM O’Garra A. The effect of antigen dose on CD4+ T helper cell phenotype development in a T cell receptor-alpha beta-transgenic model. J Exp Med (1995) 182:1579–84. 10.1084/jem.182.5.1579 7595228 PMC2192218

[106] Constant S Pfeiffer C Woodard A Pasqualini T Bottomly K. Extent of T cell receptor ligation can determine the functional differentiation of naive CD4+ T cells. J Exp Med (1995) 182:1591–6. 10.1084/jem.182.5.1591 7595230 PMC2192213

[107] Rogers PR Croft M. Peptide dose, affinity, and time of differentiation can contribute to the Th1/Th2 cytokine balance. J Immunol (1999) 163:1205–13. 10415015

[108] Milner JD Fazilleau N McHeyzer-Williams M Paul W. Cutting edge: lack of high affinity competition for peptide in polyclonal CD4+ responses unmasks IL-4 production. J Immunol (2010) 184:6569–73. 10.4049/jimmunol.1000674 20495070 PMC2930602

[109] Fazilleau N McHeyzer-Williams LJ Rosen H McHeyzer-Williams MG. The function of follicular helper T cells is regulated by the strength of T cell antigen receptor binding. Nat Immunol (2009) 10:375–84. 10.1038/ni.1704 19252493 PMC2712297

[110] Leddon SA Sant AJ. The peptide specificity of the endogenous T follicular helper cell repertoire generated after protein immunization. PLoS One (2012) 7:e46952. 10.1371/journal.pone.0046952 23077537 PMC3471970

[111] Keck S Schmaler M Ganter S Wyss L Oberle S Huseby ES Antigen affinity and antigen dose exert distinct influences on CD4 T-cell differentiation. Proc Natl Acad Sci U S A (2014) 111:14852–7. 10.1073/pnas.1403271111 25267612 PMC4205596

[112] Baumjohann D Preite S Reboldi A Ronchi F Ansel KM Lanzavecchia A Persistent antigen and germinal center B cells sustain T follicular helper cell responses and phenotype. Immunity (2013) 38:596–605. 10.1016/j.immuni.2012.11.020 23499493

[113] Fahey LM Wilson EB Elsaesser H Fistonich CD McGavern DB Brooks DG. Viral persistence redirects CD4 T cell differentiation toward T follicular helper cells. J Exp Med (2011) 208:987–99. 10.1084/jem.20101773 21536743 PMC3092345

[114] Harker JA Lewis GM Mack L Zuniga EI. Late interleukin-6 escalates T follicular helper cell responses and controls a chronic viral infection. Science (2011) 334:825–9. 10.1126/science.1208421 21960530 PMC3388900

[115] Luthje K Kallies A Shimohakamada Y Belz GT Light A Tarlinton DM The development and fate of follicular helper T cells defined by an IL-21 reporter mouse. Nat Immunol (2012) 13:491–8. 10.1038/ni.2261 22466669

[116] Pepper M Pagan AJ Igyarto BZ Taylor JJ Jenkins MK. Opposing signals from the Bcl6 transcription factor and the interleukin-2 receptor generate T helper 1 central and effector memory cells. Immunity (2011) 35:583–95. 10.1016/j.immuni.2011.09.009 22018468 PMC3208313

[117] Hale JS Youngblood B Latner DR Mohammed AU Ye L Akondy RS Distinct memory CD4+ T cells with commitment to T follicular helper- and T helper 1-cell lineages are generated after acute viral infection. Immunity (2013) 38:805–17. 10.1016/j.immuni.2013.02.020 23583644 PMC3741679

[118] Choi YS Yang JA Yusuf I Johnston RJ Greenbaum J Peters B Bcl6 expressing follicular helper CD4 T cells are fate committed early and have the capacity to form memory. J Immunol (2013) 190:4014–26. 10.4049/jimmunol.1202963 23487426 PMC3626566

[119] Gratz IK Campbell DJ. Organ-specific and memory treg cells: specificity, development, function, and maintenance. Front Immunol (2014) 5:333. 10.3389/fimmu.2014.00333 25076948 PMC4098124

[120] Hale JS Ahmed R. Memory T follicular helper CD4 T cells. Front Immunol (2015) 6:16. 10.3389/fimmu.2015.00016 25699040 PMC4313784

[121] Hori S. Lineage stability and phenotypic plasticity of Foxp3(+) regulatory T cells. Immunol Rev (2014) 259:159–72. 10.1111/imr.12175 24712465

[122] Lee YK Mukasa R Hatton RD Weaver CT. Developmental plasticity of Th17 and Treg cells. Curr Opin Immunol (2009) 21:274–80. 10.1016/j.coi.2009.05.021 19524429

[123] Goon PK Igakura T Hanon E Mosley AJ Barfield A Barnard AL Human T cell lymphotropic virus type I (HTLV-I)-specific CD4+ T cells: immunodominance hierarchy and preferential infection with HTLV-I. J Immunol (2004) 172:1735–43. 10.4049/jimmunol.172.3.1735 14734756

[124] Douek DC Brenchley JM Betts MR Ambrozak DR Hill BJ Okamoto Y HIV preferentially infects HIV-specific CD4+ T cells. Nature (2002) 417:95–8. 10.1038/417095a 11986671

[125] Saunders BM Britton WJ. Life and death in the granuloma: immunopathology of tuberculosis. Immunol Cell Biol (2007) 85:103–11. 10.1038/sj.icb.7100027 17213830

[126] Litman GW Rast JP Fugmann SD. The origins of vertebrate adaptive immunity. Nat Rev Immunol (2010) 10:543–53. 10.1038/nri2807 20651744 PMC2919748

[127] Gerlach C van Heijst JW Swart E Sie D Armstrong N Kerkhoven RM One naive T cell, multiple fates in CD8+ T cell differentiation. J Exp Med (2010) 207:1235–46. 10.1084/jem.20091175 20479114 PMC2882844

[128] McMichael AJ Phillips RE. Escape of human immunodeficiency virus from immune control. Annu Rev Immunol (1997) 15:271–96. 10.1146/annurev.immunol.15.1.271 9143689

